# Foodborne Botulism in the Republic of Georgia

**DOI:** 10.3201/eid1009.030806

**Published:** 2004-09

**Authors:** Jay K. Varma, Guram Katsitadze, Maia Moiscrafishvili, Tamar Zardiashvili, Maia Chokheli, Natalia Tarkhashvili, Ekaterina Jhorjholiani, Maia Chubinidze, Teimuraz Kukhalashvili, Irakli Khmaladze, Nelli Chakvetadze, Paata Imnadze, Jeremy Sobel

**Affiliations:** * Centers for Disease Control and Prevention, Atlanta, Georgia, USA;; † National Center for Disease Control, Tbilisi, Republic of Georgia

**Keywords:** Botulism, Food, Preserved, Paralysis, Georgia (Republic), Surveillance, Food Poisoning, research

## Abstract

Foodborne botulism is a potentially fatal, paralytic illness that can cause large outbreaks. A possible increase in botulism incidence during 2001 in the Republic of Georgia prompted this study. We reviewed surveillance data and abstracted records of patients with botulism who were hospitalized from 1980 to 2002. During this period, 879 botulism cases were detected. The median annual incidence increased from 0.3 per 100,000 during 1980 to 1990 to 0.9 per 100,000 during 1991 to 2002. For 706 botulism patients hospitalized from 1980 to 2002, 80% of their cases were attributed to home-preserved vegetables. Surveillance evaluation verified that botulism incidence varied greatly by region. Georgia has the highest nationally reported rate of foodborne botulism in the world. A strategy addressing individual behaviors in the home is needed to improve food safety; developing this strategy requires a deeper understanding of why botulism has increased and varies by region.

## Abstract 2

In the Republic of Georgia, high rates of foodborne botulism are largely caused by home-preserved vegetables.

Botulism is a severe, paralytic illness caused by toxins of the spore-forming, gram-positive rod *Clostridium botulinum*. Illness is characterized by cranial nerve dysfunction and symmetric descending flaccid paralysis, which may result in death from respiratory failure (*1*). Foodborne botulism, the most common form, is caused by eating food containing preformed botulinum toxin. Because *C. botulinum* is ubiquitous in the environment, spores routinely contaminate food and survive standard cooking practices that do not exceed 100°C. *C. botulinum* cells produce botulinum toxin only under unique conditions: an anaerobic environment, nonacidic pH, low salinity, and high water activity (a critical factor influencing shelf-life) (*2*). Home-preserved foods often attain these conditions and, therefore, present a high risk for botulism when spores survive the preservation process.

Though uncommon, botulism is a public health problem for a number of reasons (*3*). First, one contaminated food product can rapidly make a large number of people critically ill; even a single case represents a public health emergency. Second, botulism is highly preventable with proper techniques for preserving and preparing food. And third, the potential for intentional botulinum toxin release into food, water, or air obligates public health officials to gather more data about botulism for bioterrorism preparedness (*4*).

In 2001, public health officials in the Republic of Georgia (est. 2002 population 4.4 million persons) became concerned about a possible increase in the incidence of botulism. A small mountainous country in the South Caucasus bordering the Black Sea, Georgia gained independence in 1991. Although once a prosperous Soviet republic, Georgia now has a per capita annual income of US$591, making it one of the poorest countries in the world (*5*). Its public health system, moreover, has been challenged by massive outbreaks of vaccine-preventable diseases, declining sanitation, and an inability to fund its healthcare system (*6**–**8*).

At the time of this study, it was not known whether the reported increase in botulism incidence was real and what the possible causes were. To assess the magnitude of botulism in Georgia, we reviewed existing surveillance data and performed case finding and active surveillance in hospitals and public health offices. To further characterize the epidemiology of botulism, we abstracted records of botulism patients hospitalized from 1980 to 2002.

## Methods

### National Public Health Surveillance

Georgia's National Center for Disease Control (NCDC) conducts passive surveillance for botulism. Physicians are required by law to report all suspected cases of botulism to local epidemiologists who, in turn, are required to report to region-level epidemiologists and to NCDC. Administratively, Georgia is divided into 10 regions, 2 major cities, and 2 autonomous republics. The two autonomous republics, South Ossetia and Abkhazia, are not under public health surveillance.

### Medical Records Review

We visited hospitals in cities and regions of Georgia that reported at least one botulism case from 1980 to 2002. At each hospital, medical records were sorted by discharge diagnosis. A patient was considered to have botulism if medical records indicated that this was the final diagnosis. For each patient, a trained epidemiologist completed a standardized data abstraction form that included patient demographics, illness history, and clinical characteristics. A random sample of patient records (13%) was audited by a staff physician at the Tbilisi Infectious Pathology Center, which supplied most of the patients for this study, to confirm the accuracy of abstracted data; all paper abstraction forms and electronic records were compared to assess accuracy of data entry.

Outbreaks were not documented separately in national surveillance. To account for clustering of cases from common food sources, we defined an outbreak as two or more patients who were documented in the medical chart as being part of an outbreak; who had hospital admission dates no more than 3 days apart; and who had identical suspect food sources, town of residence, and hospital of admission. A botulism event was defined as an outbreak or as a sporadic case (i.e., an individual case not associated with other cases). When performed, diagnostic testing on food or human specimens was conducted at NCDC by using the standard mouse bioassay for detecting botulinum toxin (*9*).

### Surveillance System Evaluation

Because our analysis demonstrated marked geographic variation in incidence, we performed retrospective case finding and active surveillance for botulism in two parts of Georgia that reported no cases from 1980 to 2001: the city of Poti, estimated 2002 population 46,000, and the region of Samegrelo, estimated 2002 population 405,500. For retrospective case finding in these two parts of Georgia, we interviewed local epidemiologists to identify botulism cases that were not reported to NCDC from 1996 to 2002. We also interviewed physicians working at the main outpatient clinics and on inpatient infectious diseases, critical care, and neurology wards. At hospitals, we reviewed medical records to identify suspected botulism cases among patients diagnosed with neurologic syndromes from 1996 to 2002. We also reviewed pharmacy records at these hospitals to determine whether botulinum antitoxin, which is widely available, was ever administered to a patient. For active surveillance, we telephoned epidemiologists and hospital-based physicians every month from April through December 2002 to ascertain whether any new cases of botulism occurred in these two parts of Georgia.

### Home Visits

To learn more about practices in the home, we visited 14 homes in different regions of Georgia. We interviewed families about food preservation practices. We purchased tomatoes, peppers, and cucumbers at local markets, and asked persons who routinely preserve such vegetables to demonstrate this process for us.

### Ethics Review

The studies reported in this manuscript were reviewed by the human subjects committees at the Centers for Disease Control and Prevention (Atlanta, Georgia, USA) and at NCDC (Tbilisi, Georgia).

## Results

### National Surveillance Data

**Figure 1:**
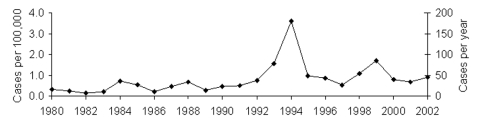
Number of botulism cases and cases per 100,000 persons in Georgia, 1980–2002. Data are derived from routine, passive national surveillance. Data are presented as one trend line because the incidence and absolute case count trend lines are indistinguishable.

From 1980 to 2002, we found 879 cases of botulism reported in Georgia. In 2002, a total of 39 cases were ascertained for an incidence of 0.9 per 100,000 persons (Figure 1). The median annual incidence increased from 0.3 per 100,000 persons (median case count 15), during 1980 to 1990, to 0.9 per 100,000 persons (median case count 41), during 1991 to 2002. The incidence was highest in 1994 (3.6 per 100,000 persons) when 173 persons became ill at a wedding from eating contaminated fish. Fifty-eight deaths were attributed to botulism, for an average death rate of 7%. The case-fatality rate ranged from 0% (several years) to 18% (in 1981). The median case-fatality rate increased from 3% (1980–1990) to 7% (1991–2002).

### Medical Records Review

We identified medical records for 706 patients with a diagnosis of botulism from eight hospitals in five regions of Georgia. Though patients resided in eight regions of Georgia, 90% of patients were initially hospitalized at or transferred to one Tbilisi hospital, which serves as the national referral hospital for botulism. Because national surveillance data consisted of only summary statistics and lacked personal identifiers, we could not determine whether the 706 patients identified through hospitals represent a subset of the total reported in surveillance (879) or all hospitalized, diagnosed botulism case-patients in Georgia.

The median age of patients was 34 years (range 1–90). Three hundred fifty-five (50%) were female. Patient ethnicities included Georgian (73%), Azerian (11%), Armenian (10%), and Russian (5%). The median age, sex, and ethnicity of patients was similar to that of the general population, except for the proportion of Azerian patients, which was almost twice as large as the proportion of Azerians living in Georgia in 1989 and 2002 (*10*). Fifty-four (8%) patients died during hospitalization.

**Figure 2:**
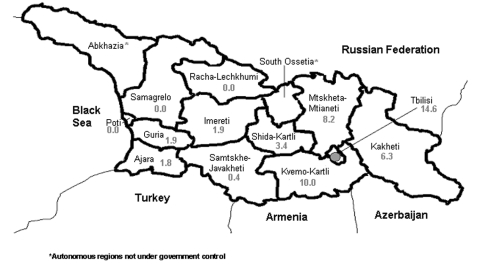
umulative incidence of botulism events by region of residence, per 100,000 persons, in Georgia, 1980–2002. Outbreaks and sporadic cases are counted as one event. Data are derived from review of medical records at hospitals. For comparison, the cumulative incidence rate of botulism events for the country was 6.7 per 100,000. Asterick indicates autonomous regions not under government control.

We identified 329 botulism events: 154 outbreaks involving 531 persons (median 3 persons per outbreak; range 2–83) and 175 sporadic cases. Medical records were available for 83 of the reported 173 patients from the 1994 outbreak. The eastern part of the country, particularly the area surrounding Tbilisi, had the highest incidence rate (Figure 2). No botulism events were identified in three areas of the country: the city of Poti and the regions of Samagrelo and Racha-Lechkhumi.

All botulism events were suspected or confirmed to be foodborne. The most commonly implicated food was home-preserved vegetables, accounting for 261 (80%) events. Other implicated foods included smoked fish (12%) and smoked meat (2%). Among events involving home-preserved vegetables, the most commonly implicated vegetables were tomatoes (15%), peppers (15%), celery leaves (13%), eggplant (13%), and combinations of vegetables (13%). The proportion of cases attributed to vegetables did not vary significantly across years or regions. Home-preserved vegetables were implicated more frequently among patients of non-Georgian ethnicity (83%) than among patients of Georgian ethnicity (64%) (p < 0.01). Botulism events varied markedly by season; most occurred in the winter (55%) or spring (23%), compared to summer (10%) or fall (12%). The proportion of events attributable to home-preserved vegetables was almost two times greater in the winter (86%) than in the summer (44%) (p < 0.01).

A specimen from either a patient or food was collected in 116 (35%) botulism events. Of events tested, 20 (17%), involving 75 persons, had botulinum toxin detected. Among these 20 events, type B toxin was identified in at least one specimen from 17 events (85%) (3 from blood, 13 from food, 1 from vomitus), type E in 2 events (10%) (1 from blood, 1 from food), and type A in 1 event (5%) (food). Home-preserved vegetables were implicated in all 17 events in which toxin type B was identified. Smoked fish was implicated in the one toxin type A event and smoked fish and home-preserved tomatoes in the two toxin type E events.

### Surveillance System Evaluation

Retrospective case finding in the city of Poti and region of Samegrelo, both of which previously reported no cases, identified three unreported, possible cases of botulism from 1996 through 2002: one involved incorrect reporting of the site of residence, the second involved failure of a physician to report, and the third involved failure of an epidemiologist to report. In the last two cases, neither patient reported eating high-risk food, neither was treated with antitoxin, and neither was given a final diagnosis of botulism by the treating physician. Review of antitoxin distribution records did not identify any patients treated with antitoxin from 1996 to 2002. Active surveillance for botulism cases did not identify any new cases from April to December 2002 in Poti and Samegrelo.

### Home Visits

We observed that the home preservation process varied between homes, but the sequence of events was common. We also found that many families inadequately sterilize equipment, conserve vegetables uncooked, fail to use pressure cookers, store vegetables for months to years, eat preserved vegetables uncooked, and, out of necessity, occasionally eat food that looks and smells spoiled. Salt, sugar, vinegar, and spices are frequently added but usually in small amounts.

## Discussion

The incidence of botulism in Georgia has been high since 1980, has increased threefold since Georgia gained independence in 1991, and varies considerably across the country. Home-preserved vegetables and smoked fish are the most commonly recognized sources of botulism.

Georgia has the highest nationally reported rate of foodborne botulism in the world. Few countries report botulism incidence. Of those that do, the highest reported rate outside of Georgia is from Russia, which reported 501 cases in 1998, an incidence of 0.3 per 100,000 (*11*). In 2001, the incidence in the United States was 0.01 per 100,000, although the rate in the state of Alaska was 1.6 per 100,000 (*12*). In the European Union, rates are all <0.1 per 100,000 (*13*).

Georgia's surveillance system works surprisingly well. Our evaluation in two low-incidence areas of Georgia leads us to believe that the geographic variation in incidence found through medical record review cannot be entirely explained as an artifact of surveillance. Using the annual incidence rate of 0.9, we would expect approximately four cases per year in Poti and Samegrelo. We did find three possible cases, but two were never given a final diagnosis of botulism. No cases were later identified through active surveillance. The incidence of disease in those areas, as well as across Georgia, may be underestimated because patients with mild symptoms fail to seek care, or patients with severe illness die before receiving medical attention. Economic collapse in the 1990s led the Georgian government to privatize the previously state-run healthcare system. Consequently, many persons cannot afford the cost of physician visits or hospital stays, and many Georgians may be unaware that botulism is one of the few medical conditions treated at public expense (*14*).

Our study has several limitations. Our evaluation of the surveillance system was limited by the use of unstructured interviews of physicians and epidemiologists. Similarly, we were unable to quantify the sensitivity of surveillance because we could not identify nonhospitalized botulism patients, and we did not have patient identifiers to link surveillance data to hospital medical records. Our medical records review included few laboratory-confirmed cases, but a separate analysis of the clinical features of botulism patients leads us to believe that misclassification was unlikely (*15*). Residence was obtained from the medical charts, but we do not think this is a source of bias. Many patients from outside Tbilisi sought care at facilities close to their residence and had records indicating where they were first evaluated. The geographic differences in incidence were also reflected in reporting by local public health agencies.

Even though presumably acidic vegetables (e.g., tomatoes) are frequently used, the preservation process in Georgia remains dangerous. In the absence of sterilization and pressure cooking, *C. botulinum* spores can survive and will elaborate toxin in solutions that are relatively neutral with low salt and sugar content. Failure to heat food before eating increases the risk further (*1*). Households probably conserve vegetables because of the absence of inexpensive, readily available, industrially preserved food, particularly in the winter, when fresh vegetables are prohibitively expensive. Dependence on home-preserved vegetables is likely to have increased since 1991 because of economic collapse, including the closure of virtually all commercial canning factories in Georgia (*16*). Other factors may include cultural preferences and inadequate awareness of attendant risks. Changing practices will be difficult because the intervention needs to be home-based and must account for the various forces that compel persons to eat improperly preserved food.

Why the rate of botulism has increased dramatically remains unclear. Poverty likely drives more persons to conserve food; lack of reliable energy sources, clean water, and cooking supplies makes food preservation practices riskier; and food shortage compels persons to rely on preserved food for a larger proportion of their diet. Poland historically reported high rates of botulism, but as economic conditions and food production improved in the 1990s, the incidence of botulism declined dramatically from 0.9 per 100,000 in 1990 to 0.2 per 100,000 in 1998 (*17*).

Wide geographic differences in incidence make us suspect that some parts of the country do not conserve food as frequently or may have developed safer techniques. These regional differences may hold the key to a successful intervention. Definitive interventions, such as developing a commercial canning industry or distributing pressure cookers, are impractical at this time because Georgia's economic infrastructure has collapsed. Instead, we may be able to prevent botulism by identifying culinary, cultural, and social factors that keep the incidence low in some parts of the country and translating those findings into a public health message for the high-incidence areas. For now, Georgia's public health message is that persons should thoroughly heat home-conserved vegetables and that medical care for botulism is free. An effective, inexpensive, culturally appropriate intervention is needed to improve food safety in Georgia.
